# Association between Female Reproductive Factors and Risk of Dementia

**DOI:** 10.3390/jcm13102983

**Published:** 2024-05-19

**Authors:** Magdalena Pszczołowska, Kamil Walczak, Weronika Miśków, Magdalena Mroziak, Gracjan Kozłowski, Jan Aleksander Beszłej, Jerzy Leszek

**Affiliations:** 1 Faculty of Medicine, Wrocław Medical University, 50-367 Wrocław, Poland; 2 Clinic of Psychiatry, Department of Psychiatry, Medical Department, Wrocław Medical University, 50-367 Wrocław, Poland

**Keywords:** dementia, Alzheimer’s disease, stillbirth, abortion, late first birth age, late motherhood

## Abstract

Women have an over 50% greater risk of dementia than men, which is a main topic of much research. This review aims to investigate the impact of a woman’s reproductive history on dementia risk. The consequences of stillbirth are long-term health and psychosocial problems for women. Because of the awareness of an endangered pregnancy, many parents experience deep anxiety and stress in subsequent pregnancies. There are contradictory conclusions from research about abortion and the risk of dementia correlation. When it comes to the late age of first birth, which is said to be above 35 years old, it was observed that older mothers have a decreased risk of dementia compared to those who gave birth in their 20s; however, being a child of the older mother is connected with a higher risk of developing dementia. Using hormonal contraception can result in decreased risk of dementia as estrogen stimulates microglia-related Aβ removal and reduces tau hyperphosphorylation. The influence of postmenopausal hormonal therapy and the duration of the reproductive period on developing dementia remains unclear. Although female disorders like endometriosis and polycystic ovary syndrome are reported to increase the risk of dementia, the research on this topic is very limited, especially when it comes to endometriosis, and needs further investigation. Interestingly, there is no conclusion on whether hypertensive disorders of pregnancy increase the risk of dementia, but most articles seem to confirm this theory.

## 1. Introduction

Biological changes and pregnancy events are obvious examples of life events that have a huge impact on a woman’s life and health. High parity and extremes of age at first birth have been linked with increased dementia risk in women, with exposure to pregnancy-associated physiological changes proposed as an explanation [1]. The impact of a woman’s reproductive history on her risk of later dementia should be investigated [2]. The upcoming dementia epidemic will be a huge burden for the entire world, having a huge impact on the lives of patients, their caregivers, families, and entire societies. A recent study found that the history of pre-eclampsia was associated with an increased risk of dementia, particularly vascular dementia [2]. Global age-standardized prevalence and death rates for dementia are higher in women than in men [3]. Women live relatively longer than men, and the risk of developing dementia increases with age. The higher prevalence and death rates from dementia in women are undoubtedly influenced by their longevity. Female-specific reproductive factors may be able to explain these sex differences in the risk of developing dementia [4].

## 2. Characteristics of Dementia

The best characterization of dementia would be as a syndrome, with multiple causes leading to it, instead of a particular disease. Dementia characterizes any cognitive decline that intrudes into independent, everyday functioning [5]. There are many causes of dementia, which we may divide into two groups: neurodegenerative causes and non-neurodegenerative causes. In the first group, we categorize conditions that cause the degeneration of neurons, a non-reversible process. Those conditions are Alzheimer’s disease (AD) and related dementias (ADRD), such as Lewy body, frontotemporal (FTD), vascular, limbic-predominant age-related TDP-43 encephalopathy (LATE), and mixed etiology dementia. Neurodegenerative diseases have underlying proteinopathies, e.g., amyloid-beta, PHF-tau, alpha-synuclein, and TDP-43 pathology. Vascular pathologies include tissue injury with or without vessel disease [6]. In the second group, we include, for example, endocrine and metabolic causes [7]. Other causes may be carbon monoxide (CO) poisoning [8], hepatic or portal–systemic encephalopathy, leukodystrophies [7], Wilson’s disease [9], and chronic kidney disease (CKD) [10]. Cognitive decline may also be caused by nutrition deficiency [11] or infectious diseases such as prions, herpesvirus, encephalitis and meningitis, and dementia associated with AIDS, Lyme disease [12], or COVID-19 [13]. There are also many studies proving that dementia may also be caused by intracranial masses [14,15] or traumatic brain injury [16]. Chemotherapy is also mentioned as a cause of disease [7]. Even profound depression and/or chronic anxiety are said to be one of the causes of dementia [17,18], or even drugs used in psychiatry like benzodiazepines [19]. There may also be radiation [20] and heavy metals exposure [21] listed as dementia causes. One of the causes repeatedly mentioned in this work is stress. This is why it is elaborately explained in the following paragraph. It is widely documented that stress disorders are associated with dementia. Although many works observed a higher risk of dementia after long-term exposure to stress, the underlying mechanism is still mostly hypothesized. The best-described theory is hippocampal atrophy as a result of stress [22]. The hippocampus plays a significant role in learning and memory. It is a structure that presents a high concentration of corticosteroid receptors [23]. In the face of stress, two stress axes are involved in response. The first, the sympathetic–adrenal–medullary axis, secretes epinephrine. The other, the HPA axis, results in the secretion of glucocorticoids, mainly cortisol [24]. MRI showed that changes in adrenal secretory pattern result in reductions in hippocampal volume [23] and, as a result, cognitive decline. The other theory claims that stress-caused neuroinflammation may increase dementia risk. Due to chronic stress, pro-inflammatory cytokines are produced. There is a hypothesis that neuroinflammation may be mediated by glucocorticosteroids, which cause leukocyte migration in the brain. Neuroinflammation disrupts proliferation, migration, and differentiation of neural stem cells, which leads to impairment in memory abilities [24].

Diagnosing dementia is not an easy task because the disease usually develops slowly, and the first symptoms may be difficult to notice. However, as the disease develops, the patient starts to have serious difficulties in everyday life [25]. Then, there should be a cognitive and neurologic examination conducted [26]. Knowledge of patients’ daily management ability may be obtained with the use of a questionnaire, which should be filled out by the patient and their caregiver [25]. Another helpful tool is the Montreal Cognitive Assessment (MoCA) 30-point test covering eight cognitive domains [27]. The Mini-Mental State Examination (MMSE) is also a very commonly used worldwide 30-point test [28].

Recent medical advances allowed for using biomarkers in diagnosing dementia, for example, fluorodeoxyglucose (FDG). If asymmetric, bilateral temporal–parietal hypometabolism on FDG is detected, it may be helpful in AD diagnosis [29]. In positron emission tomography (PET), biomarkers include 18F-florbetapir or technetium-99m-hexamethyl propylene amine oxime (99mTc-HMPAO) [30]. Genetic testing may be used in searching for rare autosomal dominant forms of dementia like presenilin gene mutations [31]. Neuroimages obtained thanks to magnetic resonance imaging (MRI) or computer tomography (CT) help to visualize atrophy of the brain structures [32,33,34].

Dementia in patients younger than 65 years old is called young-onset dementia. For those aged 45–64 years, the prevalence was 98.1 per 100,000 [35]. In differential diagnosis, late-onset forms of neurodegenerative diseases in children must be taken into account [36].

Mild cognitive impairment (MCI) may be a benign stage of dementia. It may be divided into amnestic and non-amnestic. MCI should not be a synonym for age-related cognitive decline because it exceeds this term [37].

## 3. Stillbirth and Risk of Dementia

Stillbirth is a common and devastating pregnancy complication. Stillbirths are a huge public health challenge and a sensitive indicator of the quality of perinatal care. The UN Global Strategy for Women’s, Children’s, and Adolescents’ Health (2016-30) and the Every Newborn Action Plan (led by UNICEF and the WHO) play a major role in preventing preventable stillbirths [38]. In developed countries, the most common risk factors associated with stillbirth are alcohol consumption, pregnancy using assisted reproductive technology, smoking, non-births, advanced maternal age, obesity, pre-existing diabetes, chronic hypertension, multiple pregnancies, single status, and past obstetric history. It is difficult to investigate specific causes of stillbirths because of the lack of uniform protocols for evaluating and classifying stillbirths and the declining number of autopsies [39]. The consequences of stillbirth are long-term health and psychosocial problems for women and families, and significant economic costs for parents and health care providers [40]. In a subsequent pregnancy, parents trying for a baby may face up to a fivefold increased risk of stillbirth. Because of their awareness of the endangered pregnancy, many parents experience deep anxiety and stress in subsequent pregnancies [41]. Stress is said to be one of the dementia risk factors [42]. Many disorders associated with stillbirths are modifiable and controllable and often coexist, such as maternal infections (fraction attributed to the population: malaria 8.0% and syphilis 7.7%), dietary and lifestyle factors (each about 10%), non-communicable diseases, and maternal age over 35 years (6.7%) [43].

The risk of stillbirth increases at term in all maternal age groups, especially in older mothers. For example, a total of 674 stillbirths occurred in women aged 40 years or older, and 24.2% of them (n = 163) occurred during the term of pregnancy [44]. The ratio (95% confidence interval) of proportions of women with symptoms related to anxiety above the 90th percentile for women who had had a stillborn child compared with those who had not was 2.1 (1.2 to 3.9). As soon as possible after the diagnosis of death, it is advisable to induce labor. A calm environment in which a woman can spend as much time as she wants with her dead baby is beneficial [45]. Anxiety may be particularly associated with AD [46]. In another study, results indicated elevated short- and long-term levels of depression, anxiety, and PTSD in parents after stillbirth, compared to live-born parents [47]. More longitudinal studies are needed to increase our knowledge of how symptoms develop over time.

## 4. Abortion and Risk of Dementia

Abortion is defined as the termination of pregnancy. Usually, it is performed in the first or second trimester [48] by medication or a surgical procedure. According to the WHO, 61% of unintended pregnancies end in abortion [49].

A broad Gong et al. study of 273,240 women revealed an association between reproductive factors, such as abortion, and the risk of dementia. In comparison with women who never had abortions, ones who underwent 2 and over had a lower risk of dementia. Their hazard ratio is 0.34 (*p* < 0.001). The only factors found to influence the risk of dementia associated with abortions are lifestyle and health, but the underlying mechanism still needs to be investigated. The analysis of the relationship between stillbirth and miscarriage and the risk of dementia showed no relationship [4].

The opposite conclusion comes from studies on mice models taken by Wang et al. They revealed that there might be a relationship between cognitive performance in the elderly and repeated abortion during adulthood. Mice at the age of 15 months old who underwent abortions presented cognition impairment and showed worse functions in memory tests. Researchers found that there is a higher Aβ concentration level in the CA region of the hippocampus of mice that underwent repeated abortions [50].

An Italian retrospective study did not acknowledge abortion to be a risk factor or a protective factor for dementia. They found no significant differences in Alzheimer’s occurrence between women who previously had abortions with no significant difference in regions DA and PFC. What is more, estrous cycle, which can affect cognition, was disturbed in repeated abortion mice [51].

Studies about connections between abortion and the risk of dementia are very limited. There is some more information about the impact of incomplete pregnancies on dementia risk.

Jang et al. reviewed data about 3549 women from Korea and Greece. Women with incomplete pregnancies, containing spontaneous and induced abortions, showed half the level of AD risk to those who never had one. As abortions, spontaneous and induced, mainly occur in the first trimester, there is only a modestly upregulated serum estrogen level. If this upregulation occurs within the optimal range for neurogenesis and cognition, it may reduce the risk of Alzheimer’s disease. The number of completed and incomplete pregnancies showed no influence on the risk of mild cognitive impairment. What is more, women who never experienced an incomplete pregnancy had worse results on MMSE in comparison to women who experienced it at least once [52].

Of course, the statistical analyses carried out in these studies do not provide the basis for answering the question of whether abortion is a risk factor or a protective factor against the occurrence of dementia.

## 5. Late Age of First Birth and Risk of Dementia

The average age of birth of the first child is constantly growing in industrialized countries [53]. By postponing motherhood, are women putting themselves at risk of dementia? Or are they perhaps protecting themselves against it? Can it be assumed that longer life correlates with a higher possibility of developing dementia? Can this be connected with predicted longer life for women who gave birth after 45 years of age? Jaffe et al. have investigated maternal lifespan in women considering the age of birth of their last child. Women who became pregnant between 40 and 44 years of age had a lower mortality rate of 16% compared to their peers who were not pregnant after their 40th birthday. In the group were women who were older than 45 years old during pregnancy; this rate was lowered by 42% [54]. Is the risk of dementia higher in them due to their longer life expectancy [55]? Interestingly there is much research that proves that the late age of first birth decreases the risk of dementia. In their study, Basit et al. were looking for a correlation between the age of first birth and risk for developing dementia in their population-based cohort study of more than 4 million people. Results obtained by them showed that older first-time mothers had a decreased risk of dementia compared with women who gave birth in their 20s; however, these results do not apply to the old first-time fathers. What is more interesting is that in this study, Basit et al. proved that for both sexes, having two or more children was more beneficial in terms of dementia instead of having one child. Having one child instead of being childless was associated with slightly decreased dementia risk in men, but not in women [1]. 

The reason for these findings may be not pregnancy-related hormonal factors but more so social factors that influence reproductive factors. In a decision to start a family, education, employment, material status, and lifestyle play a crucial role [56]. Those issues affect the decision of how many children to have, or if any. Those reproductive choices influence one’s life further life, including socioeconomic status, level of stress, and lifestyle, which are known to influence dementia risk [57,58]. Becoming a parent at an early age may result in lower education and socioeconomic status, and being childless decreases family networks [59,60]. 

Interestingly, Rocca et al. have observed how maternal age birth influences AD risk in their children in the future; it was higher in the children of young mothers (15–19 years old) and older mothers (above 40). Both extreme maternal ages may affect children’s brain development and lead to some changes [61]. Similar correlations were found in the risk of schizophrenia [62]. However, this topic needs to be further investigated. 

## 6. Hormonal Contraception, Postmenopausal Hormone Therapy, and Risk of Dementia

Nowadays, more and more women use hormonal contraception. Since 1960, when the first remedy, Enovid, was introduced, the concentration of steroid hormones in the pill has decreased ten times. Hence, the risk of side effects occurring has reduced [63]. Hormonal contraception includes oral contraceptives, patches, implants, vaginal rings, intramuscular depot, and levonorgestrel intrauterine system [64]. However, hormonal contraception, despite its beneficial role in planning reproduction in women, still may cause some undesirable results. Combined oral contraceptives (COCs) have been linked with a higher risk of arterial thrombosis, myocardial infarction, or ischemic stroke [65,66]. Hormonal contraception increases the risk of thrombosis, which may lead to stroke. However, the number of strokes in young women has decreased due to the limiting of the steroid factor in the oral pills and more careful screening of the patients. The risk of stroke caused by hormonal contraception is higher in occlusive stroke; nonetheless, hemorrhagic stroke risk increases with age [67]. Other reasons for stroke in young women are coagulation disorders, pregnancy, or cancer. Lidegaard found 22 studies that compromised risk for venomous thrombosis among women who use hormonal contraceptives. Based on the research, the highest risk was for norethisterone and levonorgestrel in estrogen dose 50 μg, desogestrel or etonogestrel in estrogen dose 30–40 μg, and drospirenone in estrogen dose 20 μg [64]. 

The list of potential benefits of using hormonal contraception, in addition to the main aim, includes, for example, reduction in colorectal cancer risk and ovarian cancer, and decreased risk of dementia [68,69]. It was observed that estrogen depletion in the brain may be a significant risk factor for AD development [69]. In the postmortem studies of women’s brains who suffered from AD, there was a decreased level of estrogen found [70]. Estrogen administration may prevent AD pathogenesis [71]. It was also discovered that estrogen stimulates microglia-related Aβ removal [70]. Estrogen deficiency may be so important in the pathogenesis of dementia because this hormone takes part in glucose transport and mitochondrial function, which is disrupted in patients with cognitive decline [69]. Estrogen was also reducing tau hyperphosphorylation. An interesting beneficial influence of estrogen on cerebral vessels was also observed in preventing vascular dementia. This influence contains anti-inflammatory effects and vascular tone regulation [72]. In some studies, it was suggested that there is a correlation between the length of hormonal contraception and stronger protection against dementia; however, it is not certain, and this is why further investigation is needed. Interestingly, oophorectomy also increases the risk of dementia [73].

When it comes to postmenopausal hormonal therapy (PMHT) and its influence on dementia risk, there are contradictory opinions. PMHT was invented to relieve menopausal symptoms and decrease the risk of chronic diseases such as osteoporosis, coronary artery disease, and dementia in women. It was used by many female patients by the end of the 1990s; however, the reports started to express alarm regarding the increased risk of breast cancer, coronary heart disease, and stroke [74]. 

Since the 1940s, women have been treated with estrogens to relieve menopausal symptoms; however in the late 1970s, conjugated equine estrogens administration was linked with endometrial cancer. Subsequently, progestins were added to the PMHT. It was supposed to protect because progestin could achieve a similar effect in target tissues like progesterone [75]. The Women’s Health Initiative Memory Study (WHIMS) was the first clinical trial to investigate whether PMHT reduces dementia risk in women aged 65 or older. The study was double-masked, randomized, placebo-controlled, and long-term conducted [76]. Soon after the end of the research, the results revealed that administration of continuous combined estrogen, with or without medroxyprogesterone acetate, does not protect against dementia or cognitive decline [77]. However, recent reanalysis of the trial outcomes proves that PMHT may be beneficial for women younger than 60 years old, and the risk is increasing, to a very high value in 70 years and older patients [75]. WHIMS study has been found not truly representative due to the average age of participants, which was 72 years old, when women use hormonal replacement therapy around menopause, so in younger age [69]. Further research showed that there were not any positive or negative effects on dementia risk in connection with PMHT [16,17]. Another study revealed that 10 years of PMHT results in lower dementia risk [78]. There was a hypothesis created called window of opportunity which claims that estrogen has a beneficial influence on the neuronal and vascular systems, only when it is administered soon after ovarian estrogen depletion [79]. A recent case-control study conducted in Denmark revealed that PMHT was positively associated with the development of dementia cause dementia, late-onset dementia, and Alzheimer’s disease, but the study does not explain the underlying mechanism. It also applied to women who underwent treatment at the age of 55 years old or younger, similar to both types of treatment: continuous or cyclic [80]. On the other hand, a study conducted by Hyeavon et al. showed a decreased risk of AD and VD among female patients with depression after PMHT use. The authors point out that HRT may have a protective effect against vascular dementia due to the prevention of female sex hormone deficiency. Loss of estrogen impairs cerebral blood flow by reduced vasodilation and heightened vasoconstriction. When it comes to Alzheimer’s disease, the level of estradiol was inversely correlated with the concentration of β-amyloid, and estrogen replacement reduces the number of β-amyloid oligomers [81].

All cases should be considered separately. A doctor’s decision on prescribing PMHT should be based on potential risk factors and potential benefits. PMHT should be dissuaded from use in patients with a history of breast cancer, stroke, or thromboembolic disease [75]. Some assessing risk tools have been developed; however, they do have some limitations and may not be precise for the concrete patient. Further investigation in this area is needed. 

## 7. Reproductive Period and Risk of Dementia

A scientific study conducted by Gilsanz, Paola et al. describes in detail the relationship between dementia and period of menstruation. The study included 6137 women who provided information on age at pregnancy, hysterectomy status, and age at menopause. Among these women, the mean age at the MHC visit was 51.1 years, and 76.5 years at the start of dementia follow-up in 1996. The mean age at menarche and menopause was 13.0 and 45.1 years, respectively, and the mean duration of the reproductive period was 32.2 years. A total of 2090 women (34.1%) reported having had a hysterectomy. The mean age at menarche was 13.0 years in women who did not report a hysterectomy, the mean age at menopause was 47.4 years, and the mean duration of the reproductive period was 34.4 years. Comparing women without a diagnosis of dementia with women with a diagnosis of dementia by the end of follow-up, women with a diagnosis of dementia had an older mean age at menarche, a shorter reproductive period, an earlier mean age at menopause, and were more likely to undergo a hysterectomy. The authors hypothesized that estradiol reduces inflammation, apoptosis, and tau hyperphosphorylation so longer duration of the reproductive period may reduce dementia risk. Women with a diagnosis of dementia and women without a diagnosis of dementia showed no differences in terms of education, BMI, middle-aged hypertension, or late-life diabetes [82].

Another systematic review and meta-analysis included 22 studies (475,9764 women). There was no clear association between late menarche (≥14 vs. <14 years) and dementia. In the dose-response meta-analysis, an inverse relationship was observed between a longer duration of reproduction (≥35 vs. <35 years) and dementia. They found that later menopause and longer reproductive duration were associated with a lower risk of dementia, while the relationship for menarchal age was J-shaped. Longitudinal studies are needed to further investigate the association between lifetime estrogen exposure and the risk of dementia subtypes [83].

Another study aimed to assess the relationship between reproductive factors and the risk of cognitive impairment, including mild cognitive impairment (MCI) and dementia, in Chinese women with natural menopause. A total of 4275 women aged ≥65 years who had natural menopause were included. Reproductive factors as well as the reproductive period (=age at menopause − age at menarche) were recorded. The relationships between reproductive factors and cognitive impairment were evaluated by correlation and logistic regression analysis. The prevalence of MCI and dementia was studied to be 28.6% and 11.4% in older women, respectively. Younger age at menopause, shorter childbearing years, greater age at menarche, and greater number of pregnancies/births were correlated with poor cognition and significantly increased the risk of MCI and dementia, especially AD, DLB, and VaD [84].

## 8. Endometriosis and Risk of Dementia

Endometriosis is the presence of endometrial tissue outside of the uterine cavity [85]. It is the most common cause of women’s pelvic pain [86] that affects 10% of women of reproductive age [87,88]. Infertility and chronic pelvic pain, which are the most common symptoms of endometriosis, occur in 30–50% of women diagnosed with the disease. The etiology of endometriosis is unclear. The main theory is that endometriosis develops via retrograde menstruation and the other theories suggest the role of immune system abnormalities, coelomic metaplasia, genetic causes, lifestyle, and environmental factors [89].

Wang et al.’s study of over 100,000 participants investigated if there is a connection between endometriosis and mental disorders including dementia. They did not differentiate any particular mental diseases but examined the influence of endometriosis on psychiatric disorders generally. The study demonstrates that endometriosis patients are at over twofold higher risk of mental disorders when compared to the comparison cohort [90].

Studies suggest that endometriosis may result in inflammation and metabolic changes that also affect the brain. When the brain is affected, it presents changes in gray-matter volume and altered gene expression. Some case reports suggest that psychiatric diseases such as depression, anxiety, and bipolar disorder occur more often in patients with endometriosis [91]. The connection between the appearance of dementia and endometriosis is poorly investigated and information about it is very limited. Further studies should be conducted to investigate this topic.

## 9. Polycystic Ovary Syndrome and Risk of Dementia

Polycystic ovary syndrome is a common endocrine–gynecology condition that affects 1 in 5 women of childbearing age [92]. It is characterized by the presence of enlarged polycystic ovary morphology, hyperandrogenism, menstrual irregularities, and insulin resistance [92,93]. The etiology and pathology of PCOS are not comprehensively known, although an increased ratio of luteinizing hormone (LH) to follicle-stimulating hormone (FSH) and also increased level of gonadotropin-releasing hormone are known to be the underlying cause of the disease [93].

Studies show that women affected by PCOS are reported to be at higher risk of developing Alzheimer’s disease [94] which is the most common cause of dementia [95]. Both conditions, AD and PCOS, share several risk factors such as insulin resistance, obesity, decreased vitamin D level, and increased LH to FSH ratio [96].

**Figure 1:**
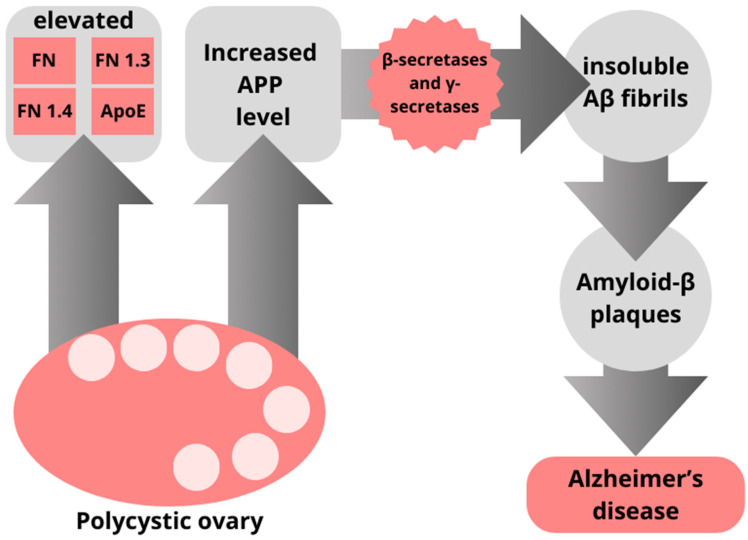
Women diagnosed with PCOS present an AD-associated protein pattern—elevated FN, FN1.3, FN1.4, and ApoE and increased APP level. APP (amyloid precursor protein) is cleaved on an amyloidogenic pathway by β-secretases and γ-secretases into insoluble Aβ fibrils. They aggregate into Amyloid-β plaques which are one of the pathomechanisms of Alzheimer’s disease.

A study of 240 women, from which 143 were diagnosed with PCOS and the rest were in the control group, revealed that PCOS is associated with elevated Alzheimer-related protein levels. It is worth mentioning that women were age-matched, but cohorts with PCOS presented higher body mass index, increased CRP level, and increased insulin resistance [94]. Obesity is known to be an AD risk factor, and inflammation and insulin resistance are some of the mechanisms underlying the pathophysiology of the disease [97]. The study revealed that women diagnosed with PCOS have an AD-associated protein pattern, which includes elevated FN, FN1.3, FN1.4, and ApoE and also increased APP level [94]. APP (amyloid precursor protein) is cleaved on an amyloidogenic pathway by β-secretases and γ-secretases to produce insoluble Aβ fibrils that aggregate into plaques that are one of the pathomechanisms of Alzheimer’s disease [98]. This was presented in the Figure 1.

A Canadian population cohort revealed that dementia associated with PCOS begins to develop 19 years sooner than dementia in women without PCOS. The study showed that the onset of dementia in women with PCOS is at a median of 43 years, while without PCOS at 62 years. The risk of developing dementia-related symptoms is 2 times higher in women affected by PCOS than controls [99].

Despite the seriousness of cognitive impairment in women diagnosed with PCOS, the research in this area is still very limited. Further studies should be conducted to better understand the occurrence of dementia and PCOS.

## 10. Hypertensive Disorders of Pregnancy (HDP) and Risk of Dementia

We can distinguish four types of disorders associated with hypertension during pregnancy; those are chronic hypertension, gestational hypertension, preeclampsia–eclampsia, and preeclampsia superimposed on chronic hypertension [100]. Postma et al. examined 145 women: 48 with no HDP, 51 preeclamptics, and 46 eclamptics. Their methods consisted of a cognitive failure questionnaire, neuropsychological tests, and Hospital Anxiety and Depression Scale. Their studies showed that women with HDP demonstrated minor slowing in motor speed, but neurocognitive disorder was not confirmed. They suggested that incoming studies should be conducted in 20–30 years [101]. The studies of the Swedish population in 2017 also did not prove a clear connection between HDP and dementia in the future [102].

However, other scientific works demonstrate different conclusions. Schliep et al.’s systematic review revealed that there is an association between hypertensive disorders of pregnancy (HDP) and risk of dementia. They found that HDP increases the risk of Alzheimer’s disease and unspecified dementia by 38%. The risk of vascular dementia among women with HDP history increases threefold [103].

Women suffering from HDP develop later conditions such as overweight, obesity, chronic hypertension, and other cardiovascular diseases. Normotensive pregnancy has a lower risk of cognitive impairment and white matter atrophy 35 years after their delivery than HDP patients [104]. Significant quantities of that atrophy occurred in the frontal lobes [105]. Women with preeclampsia, confirmed by their medical history, have more intensive calcium deposits in coronary arteries and carotid atherosclerosis, which result in hypertension, gray matter lesions, and worse cognitive abilities [106]. If patients need to use at least 2 antihypertensive drugs during pregnancy, the risk of cerebrovascular disorders is even bigger [106].

Diseases associated with dementia such as Alzheimer’s disease are often examined by magnetic resonance imagining. The brains of people suffering from dementia have impaired white matter and deterioration of blood vessels [107].

STOX1 gene is one of the most important genes responsible for trophoblast impairment, which is one of the risk factors of preeclampsia. Studies show that STOX1, as a transcription factor, influences the LRRTM3 gene, which can be found in the placenta and brain. That gene is responsible for activating the amyloid precursor protein and intensified production of amyloid beta [108]. Amyloid beta is deposited excessively in the brain and is one of the most important pathogenic factors of Alzheimer’s disease [98].

## 11. Final Remarks and Conclusions

Among many factors that contribute to the fact that women are over 50% greater risk for dementia than men, there is a need to investigate if there are factors concerning mainly the female part of society. We have presented most of our findings in Table 1. One of those factors is stillbirth, which causes stress and deep anxiety, which may negatively affect women’s mental health. What is more, in women who experience miscarriage, there is a greater risk of developing atherosclerotic disease and diabetes later in life, which are risk factors for dementia.

There were conflicting conclusions when it came to abortion as a dementia risk factor. This topic should be precisely investigated in the future to find its influence on dementia risk. In such research, it should be taken into account the different motivations of women who undergo such a procedure and their attitude towards this intervention. Perhaps distant views of the patient on this surgery, and hence her attitude towards it, may result in contrasting consequences for the patient’s mental health.

Late age of first birth correlates with decreased dementia risk; however, children of such mothers have increased dementia risk and other diseases. Pregnancy in the 40s is also a bigger threat to women’s health in total, so decisions to postpone the pregnancy due to cognitive decline prevention are precarious. However, there is no clear conclusion as to whether a long reproductive period in general increases or decreases the risk of dementia.

Using hormonal contraception is continually more and more popular, and its benefits include reduction in colorectal and ovarian cancer risk, and decreased risk of dementia. Estrogen deficiency in the brain may be a risk factor for AD development. Despite that, the influence of postmenopausal hormonal therapy on developing dementia remains unclear.

There is no clear connection between endometriosis and dementia, although there are reports that provide evidence that endometriosis increases the risk of mental disorders in general. It is for sure a topic that needs to be investigated in the future.

Another female disease, polycystic ovary syndrome, seems to increase dementia risk due to amyloid-β plaque aggregation. Some studies claim that PCOS increases the risk of dementia up to twofold.

Although most reviews confirm that hypertensive disorders of pregnancy increase the risk of dementia, there is still no proven clear connection between HDP and dementia. Women suffering from HDP develop conditions that are dementia risk factors, such as overweight, obesity, and chronic hypertension.

However, there needs to be further research conducted to fully understand those correlations and to enable new prevention and therapeutic methods.

## Figures and Tables

**Table 1:** The table summarizes how examined reproductive factors are related to the type of dementia and the underlying mechanism.

Reproductive Factor	Related Type of Dementia		Underlying Mechanism
Reproductive factors that increase dementia risk			
Stillbirth	Alzheimer’s disease		Influence of stress and anxiety
Early age of birth	Unspecified		May results in lower education and socioeconomic status that are dementia risk factors
Endometriosis	Unspecified		Unspecified, there are suggestions about inflammation and metabolic changes
Polycystic ovary syndrome	Alzheimer’s disease		Elevated Alzheimer-related proteins, insulin resistance, obesity
Hypertensive disorders of pregnancy	Alzheimer’s disease		Preeclampsia—STOX1 gene influences LRRTM3 gene which activate APP
	Vascular dementia		Unclear if HDP provokes endothelial changes or it increases the risk by sharing the same risk factors with vascular dementia
Reproductive factors that decrease dementia risk			
Late age of first birth	Unspecified		It is usually connected with higher education employment and material status, which are protective factors against dementia
Hormonal contraception	Alzheimer’s disease		Estrogen stimulates microglia-related Aβ removal and reduces tau hyperphosphorylation
	Vascular dementia		Anti-inflammatory effects and vascular tone regulation
Reproductive period	Alzheimer’s disease, Vascular dementia		Estradiol reduces inflammation, apoptosis, and tau hyperphosphorylation so longer duration of reproductive period may reduce dementia risk
Reproductive factors with uncertain influence on dementia risk			
Abortion	Decreased risk	Unspecified dementia	Lifestyle and health
	increased risk	Alzheimer’s disease	higher Aβ concentration level in the hippocampus
Postmenopausal hormonal therapy	Decreased risk	Alzheimer’s disease	Estrogen replacement reduces the number of β-amyloid oligomers.
		Vascular dementia	Estrogens prevent reduced vasodilation and heightened vasoconstriction
	Increased risk	all cause dementia, late onset dementia, Alzheimer’s disease	Unspecified
